# Blacking out

**DOI:** 10.1186/s41937-020-00052-y

**Published:** 2020-07-23

**Authors:** Yvan Lengwiler

**Affiliations:** University of Basel, Faculty of Business and Economics (WWZ) and Center for Innovative Finance (CIF), CH-4002, Basel, Switzerland

**Keywords:** COVID-19, Electricity, Seasonal adjustment, Weather data, C50, E01

## Abstract

The partial shutdown of the economy following the outbreak of the COVID-19 pandemic has highlighted the lack of measurements of economic activity that are available with a short lag and at high frequency. The consumption of electricity turns out to be a valuable proxy, if it is corrected for influences from calendar and weather. Indeed, this proxy suggests that we are currently facing one of the deepest recessions ever.

## Introduction

On February 24, 2020, the first infection with SARS-CoV-2 was detected in Switzerland. On March 5, the first patient deceased. On March 16, the government closed down a significant part of the economy and public life.

The first measure was already taken on February 28: the government issued an ordinance forbidding gatherings of more than 1000 people^1^. On March 13, it issued an ordinance with more drastic measures. It restricted travel from Italy and ordered schools to close. Gatherings of more than 100 people were restricted. Restaurants were restricted to host at most 50 guests at the same time. Only 3 days later, on March 16, the ordinance was changed and a broad shutdown of parts of the economy was initiated: all shops and open markets, restaurants, entertainment and cultural enterprises (including alpine tourism), and services with physical contact (such as hairdressers, etc.) were ordered to close. Exceptions were granted to food shops, take-away restaurants, pharmacies, hospitals (with restrictions), postal services and banks, public transportation, gas stations, social emergency institutions, public administration, and a few others^2^. Several further tightenings were ordered in the following weeks (including restrictions on travels from France, Germany, Austria, Spain, and all non-Schengen states). Notably, on March 21, public gatherings of more than five people were forbidden.

During these developments, it was obvious that the measures would have significant economic impact. But hard data on the quantitative effect was not available. GDP data are available at quarterly frequency and only with considerable delay. What the decision makers need is a rough proxy that is available with short delay and at high frequency, a proxy that can give guidance to the question: “How is the economy doing? Right now?”

Several possible proxies come to mind. For instance, traffic data (movements of cars, usage of public transportation, and transported tons of goods) could contain valuable information. Air pollution data is probably also closely connected to economic activity and is continuously monitored. The number of payments per unity of time could also be of interest. If the situation is dire, the number of insolvencies also clearly contains information about economic distress. All of these data are available long before GDP is estimated and published, and a comprehensive high frequency GDP indicator will combine most of them^3^.

One interesting candidate as a proxy of economic activity—the one I focus on in this paper—is electricity consumption. Electricity is essentially not stored at the site of consumption. Instead, electricity is largely created just in time, and to the extent it is stored, this happens in facilities of the producers. Moreover, almost all economic activity requires electricity. As a result, the consumption of electricity should be a proxy for economic activity. A testament to that is the clearly visible 7-day seasonality of electricity consumption: there is significantly less consumption on weekends than during weekdays.

The role of electricity as a means or as a measure of economic activity has long been recognized. In 1956, the Federal Reserve Board added electricity production as a component to the indicator for industrial production (Federal Reserve Board 2019). Kraft and Kraft (1978) explore the direction of causality between energy consumption and GDP, which initiated a substantial literature. In 2010, the Economist created a proxy for China’s GDP that is based on three indices, one of them being electricity (Economist 2010). Related to this is also Vernon et al. (2012), who use light emissions visible from outer space as a proxy.

Electricity consumption cannot be used as a proxy without treatment, however, because it is influenced by non-economic, exogenous factors. The most important, it turns out, is weather. In the following, I control for fluctuations that are due to weather, day of the week, Easter (and its sister holidays), and the beginning and end of daylight savings time (DST) periods. Moreover, a time fixed effect captures seasonality throughout the year that is not weather-related. The result is a series that is adjusted for calendar and weather effects. Remaining fluctuations of this series should be due to fluctuations of economic activity.

## Analysis

### Data

**Fig. 1:**
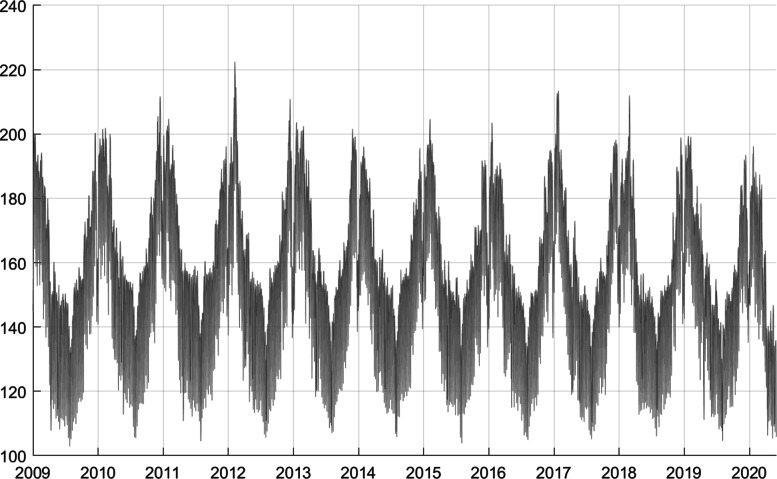
Raw data: final consumption of electrical energy in Switzerland, aggregated to daily frequency [GWh], January 01, 2009, to May 31, 2020

Electricity consumption data are available from Swissgrid^4^, the national grid company of Switzerland. I use the “final energy consumption” data which is available from January 1, 2009, at 15-min frequency to March 31, 2020 (at time of writing), see Fig. 1. The data of the last month are released around 20 days into the next month. There are also monthly data of electricity consumption covering January 1990 to December 2019^5^. I will explore those data as well.

Weather data are available from Meteoswiss. I use temperature and precipitation data collected from weather stations in the major cities, as they have good explanatory power for electricity consumption^6^.

### Specification of the regression

**Aggregation**

Data are aggregated to daily frequency by adding up the 15-min data. Care must be taken for days at which DST starts or ends. On the last Sunday of March, an hour is removed at night, and the reverse is done on the last Sunday of October. The data are denoted with *Y* and the logarithm of the data with *y*. The analysis will be performed on logarithmic data.

**Trend**

All the explanatory variables (calendar, weather) are stationary by principle^7^. To remove a trend that may be present in the electricity consumption, I compute a slow moving trend and perform the analysis on the difference from the trend. I use the Hodrick-Prescott (HP) filter because this filter has relatively good edge of sample properties. The HP trend of *y* is denoted with *y*^∗^. The non-logarithmic version is denoted with *Y*^∗^:= exp(*y*^∗^)^8^.

**Weather**

Various regressions have revealed that the heating degree variable has more explanatory power for electricity consumption than raw temperature. Heating degrees is a rough measure of the need for buildings to be heated. There is a Swiss and a US definition. I try both, but the US definition seems to do slightly better in most cases, so this is the one I use. In addition, precipitation also has some explanatory power, so this is included as well.

**Fixed calendar effects (seasonality).**

To catch the obvious seasonality, I add fixed effects to each calendar day in the year. Think of this as a panel regression where *T* is the calendar day of a year (i.e., day and month), and *N* is the year of observation. The calendar-day fixed effects are dummies for each day and month combination in *T*. The coefficients of these fixed effects trace the volatility that repeat on a yearly basis, what we normally call seasonal factor.

**Day of week.**

I use dummies for Monday to Friday. It turns out that the seasonal effect of Saturdays and Sundays is not constant across calendar months. I therefore use dummies for Saturdays and Sundays interacted with the calendar month. Moreover, reduced electricity consumption on Sundays is getting weaker over time. I therefore also use a dummy that interacts Sundays with the year.

**Moving calendar effects.**

I use a dummy for Easter which does not have a fixed position in the Gregorian calendar. Connected to Easter are several other holidays, which are also moving around in the calendar and for which I also use dummies. One of these is Ascension Day, which is 39 days after Easter and always on a Thursday. It is common for many workers to take the Friday after Ascension off, so I add another dummy for the day after Ascension.

Moreover, I add dummies for the days at which DST starts (those days are an hour shorter) and when DST ends (those days a an hour longer).

**Holidays on weekends.**

The seasonal effect of some obvious holidays (such a Christmas etc) is negative. But it turns out that the effect of lower electricity consumption due to holidays and lower consumption due to the day being part of a weekend are not simply added together. I therefore add one more dummy that interacts most important holidays with the day being a Saturday or Sunday.

**Estimation**

The regression is performed on the logarithmic difference between the data and the trend, *y*−*y*^∗^. The explanatory variables are as introduced above (weather, moving calendar effects, fixed calendar effects) and are denoted with *x*. *β* denotes the coefficients. The residuals are autocorrelated, which is corrected by adding two auto-regressive terms,

$$ y_{t} - y_{t}^{*} = \beta x_{t} + g_{t}, \qquad\text{with}\qquad g_{t} = \rho_{1} g_{t-1} + \rho_{2} g_{t-2} + \epsilon_{t}, $$

where *ε* are the innovations. We can rearrange this equation in a meaningful way,

$$y - y^{*} - \beta x = g. $$

*g* is the logarithmic *electricity gap*. It measures the relative deviation of electricity consumption from the trend after all the non-economic influences—weather and calendar effects—have been removed. It is therefore a measure of economically induced variations of electricity consumption.

The *adjusted series* is defined as

$$Z := \exp(y^{*} + g) = \exp(y - \beta x). $$

This series measures consumption (in GWh) again after all the non-economic influences have been removed, but keeping the general multi-year trend (if present).

The adjusted *R*^2^ of the regression is 99.2*%*^9^. The day of the week dummies explain 35.5*%* of the variance, the seasonal factor explains 20.2*%*, the weather data explain 11.4*%*, Easter and the holidays connected to it explain 2.2*%*, and cross-terms of weather data and seasonality explain 10.9*%*.

### Seasonal pattern

**Fig. 2:**
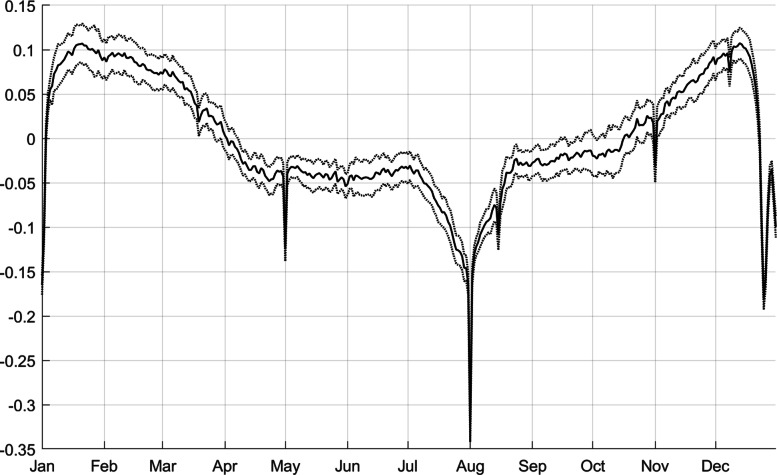
Daily seasonal factors (calendar-day fixed effects), with 95% confidence bands

Before moving on to studying the adjusted series *Z*, it may be interesting to take a look at the seasonality of electricity consumption. The seasonal structure is captured by the coefficients of the calendar-day fixed effects. I normalize these coefficients to be zero on average and call this the *seasonal factor*. This factor captures how electricity consumption is larger or smaller than average on particular days of the year. It is depicted in Fig. 2.

We see distinct downward spikes at specific dates: labor day (May 01), National Celebration Day (August 01), Mary Ascension (August 15), All Saints’ Day (November 01), Immaculate Conception (December 08), Christmas (December 24–25), and Sylvester-New Year (December 31–January 01).

### The adjusted series

**Fig. 3:**
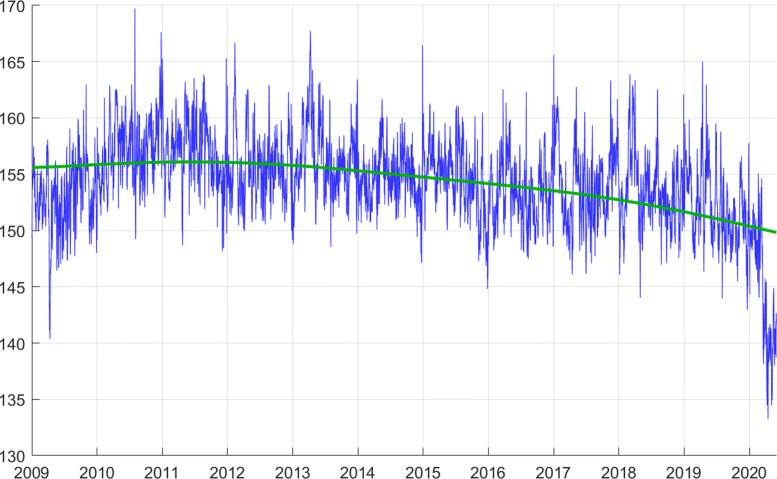
Daily data adjusted for weather, calendar effects, and seasonality (*Z*), together with the trend (*Y*^∗^)

The adjusted series *Z* tracks electricity consumption fluctuations that happen for reasons other than weather or calendar. Figure 3 depicts the adjusted *Z* and the trend *Y*^∗^.

Let us first meditate a little bit on what we see here. The trend *Y*^∗^ is roughly constant over the sample period. The adjusted series *Z* has high frequency volatility but is also essentially flat. The consumption of electric energy in Switzerland, after controlling for various non-economic influences, is essentially flat. We consume today the same amount of electricity as we did 11 years ago. Real GDP has increased by 21% in that time-frame. Population has increased by almost 10%. Yet, electricity consumption remained unchanged. This is remarkable.

## The relative electricity gap — how large is the impact of the shutdown?

### Comparing with seasonal effects

**Fig. 4:**
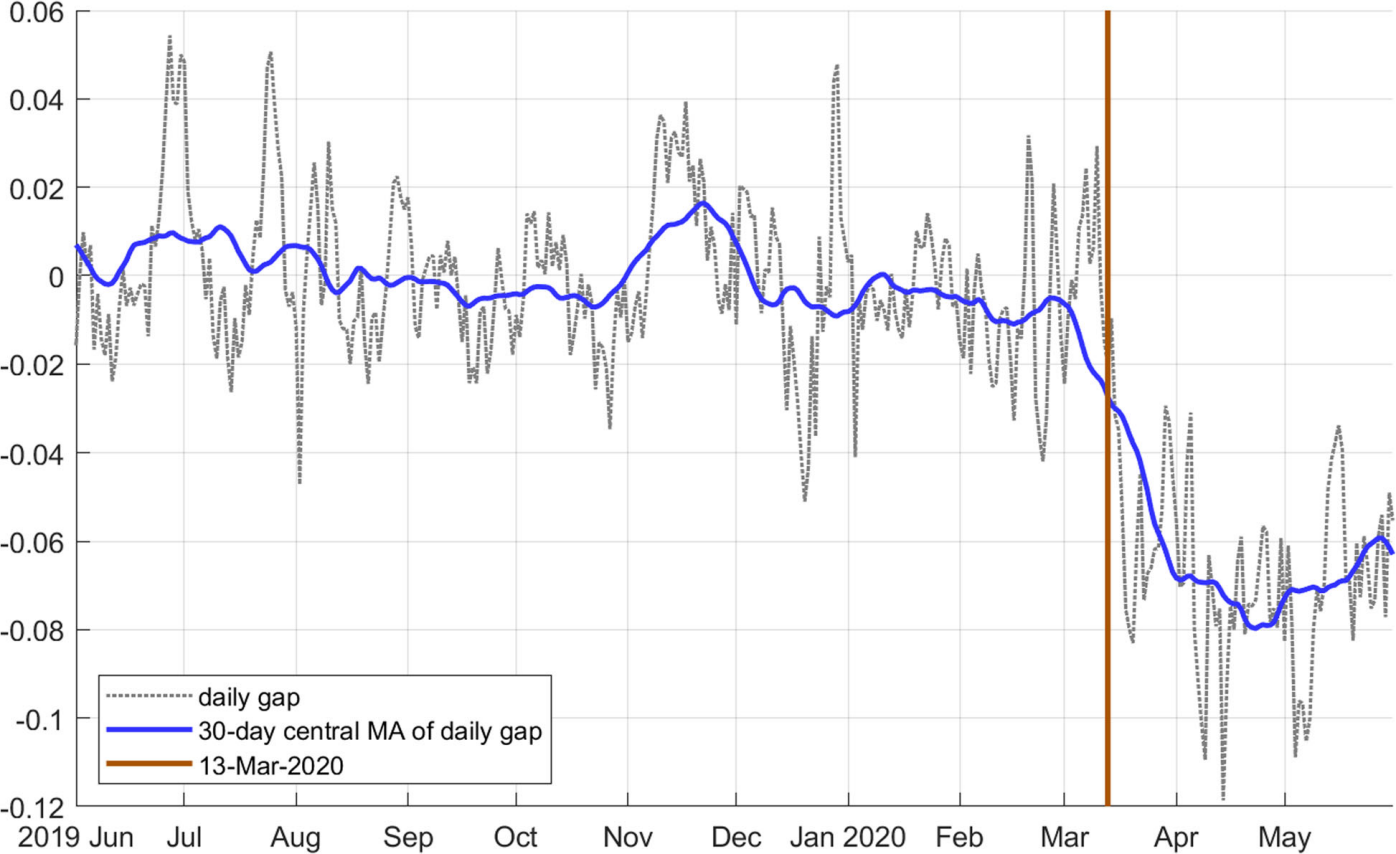
Daily electricity gap *g* (black dotted line), 30-day central moving average (blue) last 12 months

Electricity consumption is essentially flat over the sample. But this is true only until the day the shutdown came into effect. On March 13, 2020 (Friday), the first version of the strict COVID decree was initiated, and it was replaced by a much stricter version on the following Monday. We see a sharp decline of electricity consumption at and after that date. Figure 4 depicts the electricity gap *g* (the logarithmic difference between the adjusted series and the trend) for the last 12 months of the sample. We see that in the days after the shutdown came into effect, there was drop of 8.3*%*. The maximum drop of 11.8*%* was reached on April 14. The 30-day moving average dropped by 8%. This is huge in historical perspective.

The seasonal effect of August 1 is a reduction of 33%, that of Christmas is minus 18%, see Fig. 2. The quantitative effect of the shutdown is therefore about a quarter of an August 1, or half of a Christmas, but for as long as the shutdown lasts.

### Comparing with the GFC

Switzerland experienced a major recession in the fourth quarter of 2008 (− 1.9*%* quarter on quarter growth) and the first quarter of 2009 (− 1.6*%*) during the GFC. It would be interesting to see if the electricity gap picks up this major recession. Alas, daily electricity data are not available before 2009, so it may be difficult to judge if the GFC is captured by these data. The whole GFC is safely covered by the time span available at monthly frequency, however. The application of the method to monthly data is essentially the same as applied to daily data and is described in greater detail in the Appendix.

**Fig. 5:**
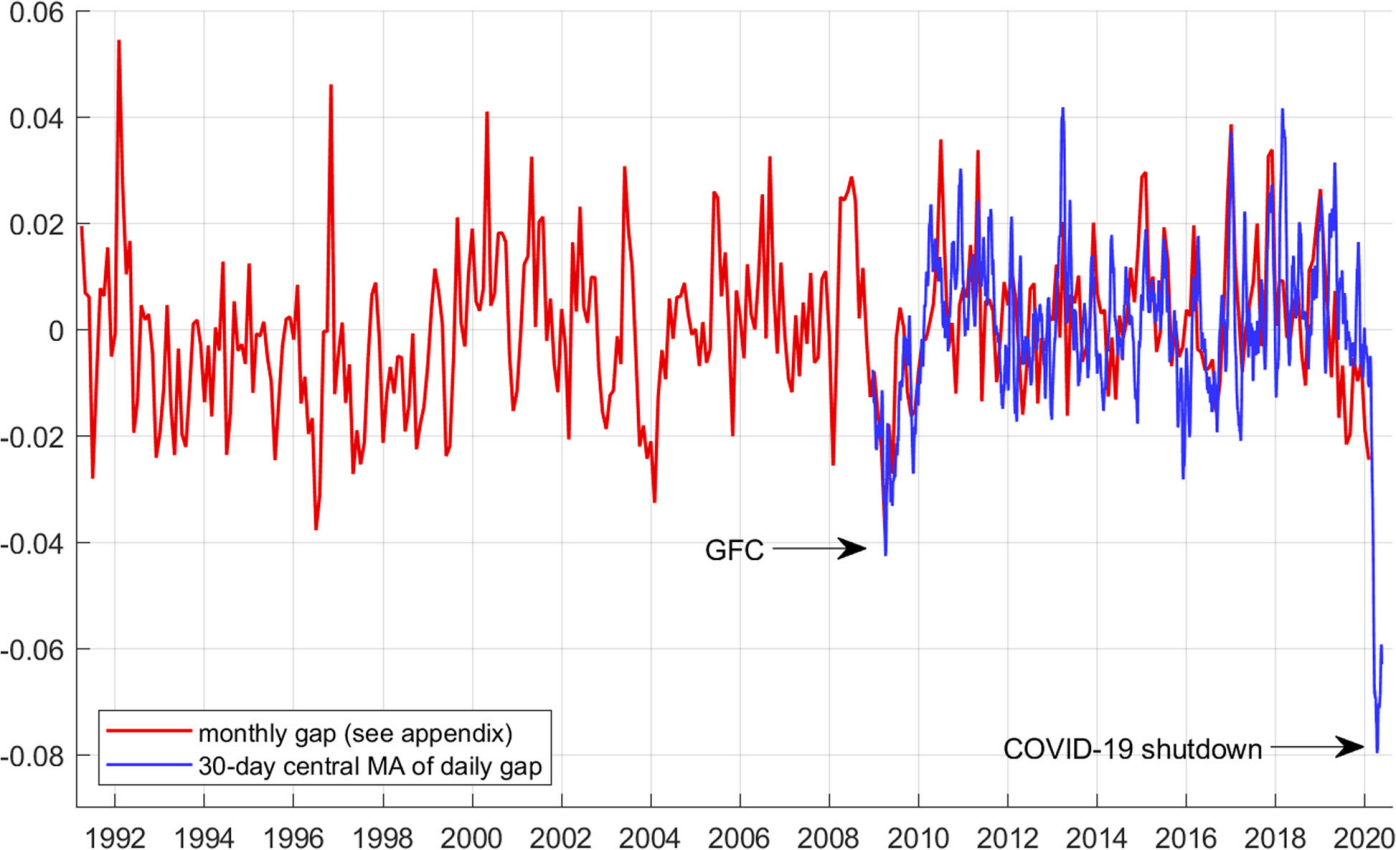
Monthly gap (*g*) in red, April 1991 to December 2019, and 30-day moving average of the daily gap in blue, January 2009 to May 2020

In the monthly electricity gap, the GFC shows up only during 2009, not before. According to the monthly gap, the GFC is estimated to be a − 3.5*%* shock in terms of electricity consumption. According to the daily gap (30-day central moving average), the shock is − 4*%* (see Fig. 5). Using the same daily measure, the COVID-19 shutdown is − 8*%*. Thus, using electricity consumption as our guide, this implies that the shutdown is a shock that is about twice as large as the GFC was.

### Progressive easing since April

Starting April 16, 2020, the government started to remove restrictions. The restrictions for hair dressers, flower shops, and a few others were the first to be removed, effective on April 27. Primary schools, general shops, libraries, restaurants (with some restrictions still in place), etc., were allowed to reopened on May 11. Church services were allowed to resume on May 28, public gatherings of up to 30 participants on May 30. On June 6, most restrictions were removed: higher schools reopened, discos and clubs (with some restrictions), as well as brothels were allowed to open, as well as casinos, campings, public swimming pools, etc., and the remaining restrictions on restaurants were eased. Moreover, political rallies and school events with up to 300 participants became possible again that day. Most borders reopened on June 15. On June 22, public gatherings of up to 1000 people were allowed and the remaining restrictions on restaurants, discos, and clubs were lifted.

Our electricity data currently ends with May 31, 2020. We therefore cover the first wave of easing measures, but not the complete reopening of the economy and social activities. The measurement shows only a very modest rebound of activity, though. It appears that electricity consumption remains subdued almost to the level it fell to at the inception of the shutdown (Fig. 4).

## Conclusion

In an emergency, speediness of information gains importance relative to accuracy. A piece of information that points in the correct direction without getting the details right is of higher value than a detailed and precise account that is available 3 months too late. GDP is a slow measure. It is not even very accurate, but it is the most accurate measure of economic activity we have. But because it is available with such a long delay, and then is typically revised with the benefit of new information later, it is of little use in a crisis such as the COVID-19 pandemic.

Several high frequency data sources could be considered to construct a rough but quickly available proxy for economic activity. Travel, cargo, air pollution, and payment activity could all be helpful, as is electricity consumption. A most useful high frequency proxy will combine the information of several such inputs.

This paper explores the use of electricity consumption for this purpose. It was born out of the need to have some indicator now. So it is not perfect. It could be improved, possibly, by evaluating in a more granular fashion the local amount of electricity that is being used and correlating this with industries that are locally present.

**Fig. 6:**
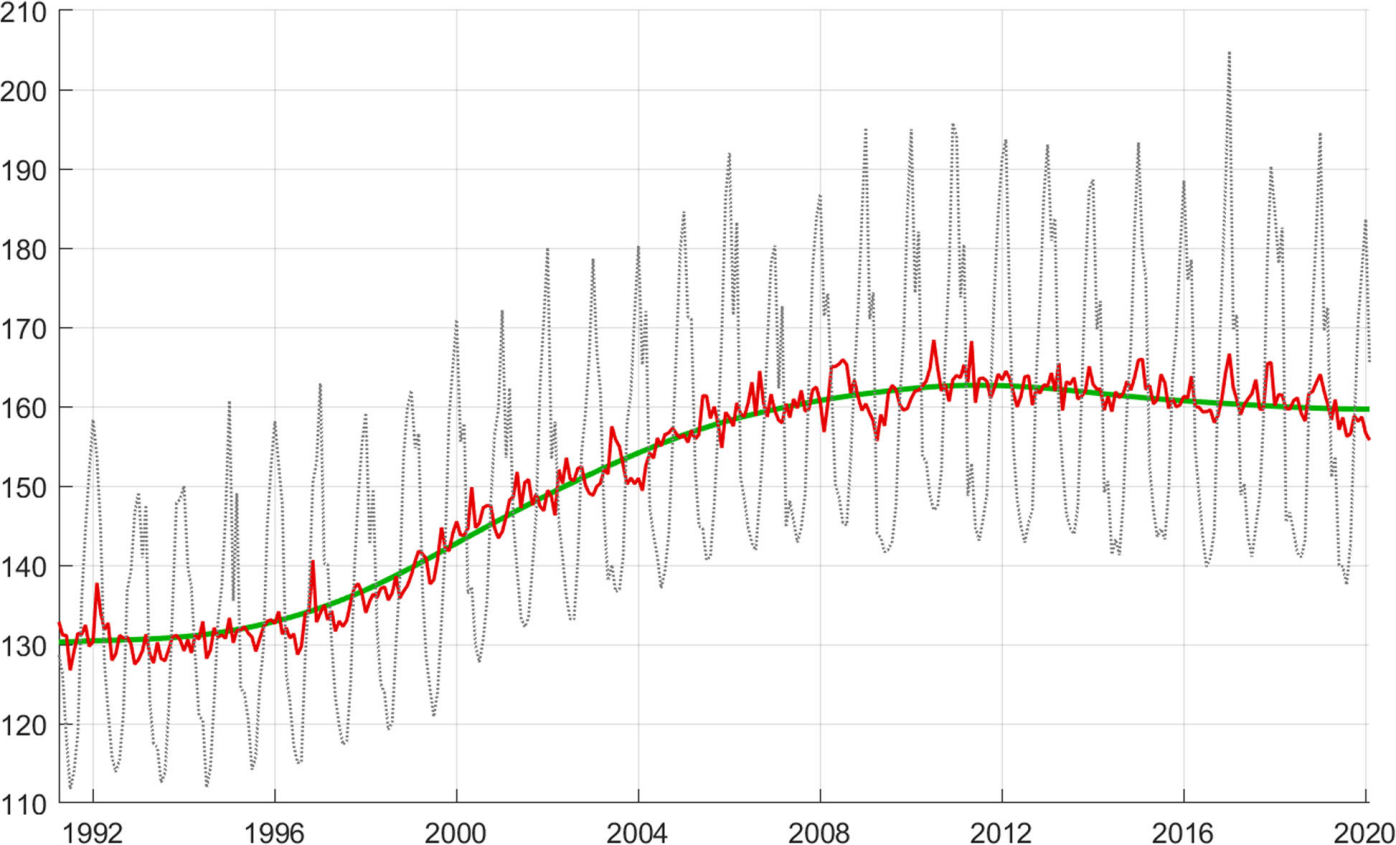
Monthly data (*Y*), adjusted series (*Z*), and HP trend (*Y*^∗^) [measured in GWh]. The original data are divided by 30 to make them roughly comparable to the daily data

The analysis has also shown that it is advisable to work with high frequency data (daily in this case), and then generate a smoothed signal from this. The resulting measure appears more informative and less volatile than if monthly data are used to begin with.

The conclusion of this paper is that the shutdown is about twice as strong as the GFC was. Comparing to major holidays, it is as strong as a quarter of the “natural shutdown” that occurs each year at August 1, or half of Christmas. But it lasts as long as the shutdown is in effect. This should give us an understanding of the economic impact of the shutdown.

The government has in the meantime decided to progressively ease the shutdown, and on June 6, most were removed. This means that the shutdown will have lasted 12 weeks. It is possible that further shutdowns may become necessary should there be flare ups of the disease. But even if this is not the case, the shutdown amounts to at least 12 weeks of a quarter of the effect of August 1, akin to 21 days of national celebration day in a row.

Of course, this takes only electricity into account. It is entirely possible that this does not fully capture all economic activities, so that this indicator is not fully meaningful. Still, gauging the size of the electricity shock, there is little doubt that the current shutdown is a shock of historical proportions and greater than anything we have seen so far in this still young century. We should brace for the longer-term impact.

## Appendix A: Using monthly electricity data and comparing with GDP

### Monthly data

There are two reasons to apply the method not just to daily but also to monthly data. One reason is to extend the period that is covered. The other reason is to check robustness and consistency of the method.

Daily (and sub-daily) data of electricity consumption are not available before 2009. Monthly data are available from January 1990 to February 2020. The weather data for one of the stations is available only from April 1991 onward on, however, so the estimation will be done from April 1991 to February 2020. The procedure that has been used for the daily data is applicable in much the same way on the monthly data.

The monthly data *Y* do show an increase between roughly 1996 to 2008 (Fig. 6). This low frequency movement is removed by taking the difference of the logarithmic data *y* from the trend *y*^∗^ (HP filter for *p*=12), and the regression is performed on *y*−*y*^∗^.

The procedure is the same before, but the regressors are a bit simpler: the average heating temperature (US definition) and precipitation in each month; the month in which Easter Monday is located^10^; the number of Mondays, Tuesdays, …, Sundays in a month; and finally, a dummy for the calendar month to capture seasonality that is unrelated to the weather. All of these explanatory variables are highly significant and the fit is again very good (adjusted *R*^2^=0.983).

### Comparing the daily and monthly adjustments

**Fig. 7:**
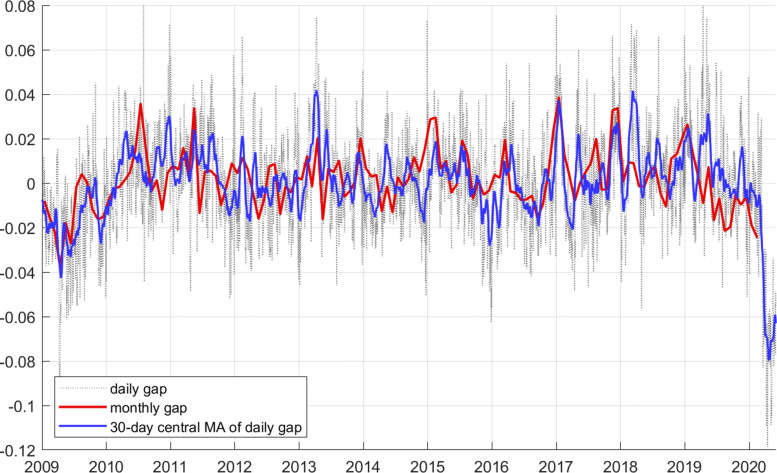
Relative electricity gap (*g*), using monthly data (red), daily data (light dotted), and the 30-day central moving average of the daily gap

The robustness check amounts to checking whether the relative electricity gap—our main indicator of economic fluctuations—computed with the monthly data provide the same signals as the relative gape derived from daily data does. Figure 7 makes that comparison. The daily gap (fine dotted line) has of course much more volatility, because it is able to capture day-to-day changes. A more comparable version is a 30-day central moving average of the daily gap, represented by the blue line. The red line is the monthly estimate. The match is not perfect, but the more substantial shocks are captured by the monthly and the daily data in much the same way. Even their sizes are very similar, although the monthly indicator appears somewhat more volatile. I conclude therefore that the two measures capture similar disturbances. I also conclude that the daily gap is more useful than the monthly gap, because a smoothed version of the daily gap produces less volatility and provides a cleaner signal for large disturbances.

### Comparing electricity gaps with GDP data

In the fourth quarter of 2008, real GDP declined (seasonally adjusted) by 1.9% from the previous quarter and an additional 1.6*%* in the first quarter of 2009. This was the deepest recession in Switzerland in the years of the GFC, and it was the deepest recession that had occurred since the oil crisis of the 1970s^11^.

**Fig. 8:**
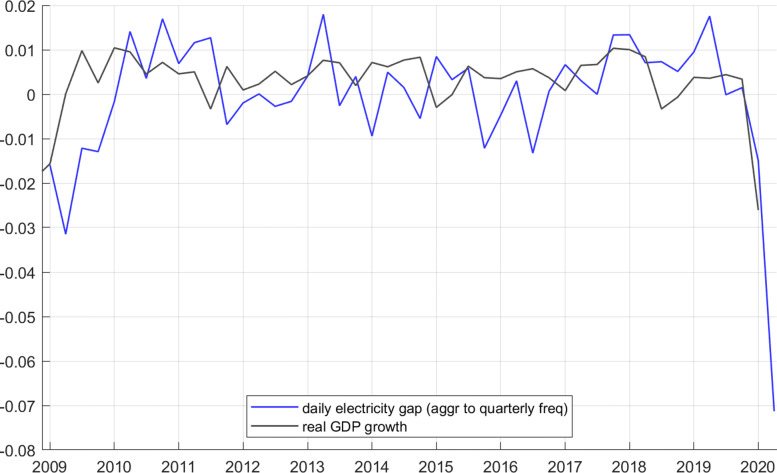
Real GDP quarterly growth rates in black, and daily electricity gap in red, aggregated to quarterly frequency

It is informative to check how the electricity gap captures this major recession. GDP data are only available at quarterly frequency. To ease comparison with the daily electricity gap, I aggregate the gap to quarterly frequency by taking the average over the days in each quarter. Figure 8 shows the result.

We see that the 2009 recession is well captured by the daily electricity gap. Smaller movements of GDP, however, seem more or less unrelated to the electricity gap. It seems that the gap is a useful signal only for rather large events.

**Fig. 9:**
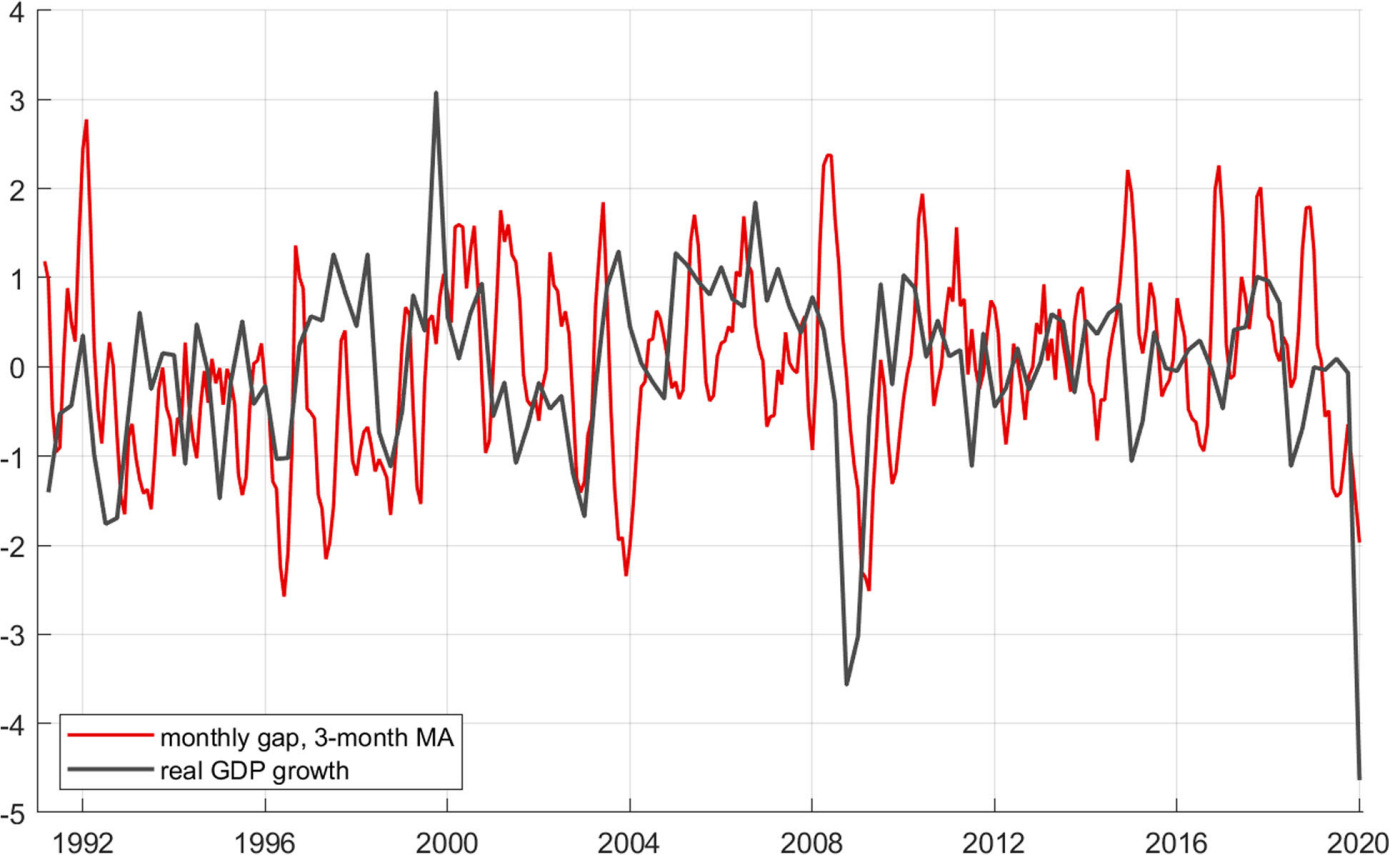
Real GDP quarterly growth rates in black, and 3-month central moving average of relative monthly electricity gap (*g*) in red. Data are normalized to have zero mean and unit volatility

Figure 9 depicts quarterly real GDP growth rates (seasonally adjusted) together with a 3-month central moving average of the monthly electricity gap. To enhance optical comparison, both series are normalized, so that they have zero mean and unit standard deviation (to correct for the different volatilities of the two series).

A visual inspection reveals that the connection between the two series is far from perfect. In particular, the electricity gap wrongly identifies a deep recession in the first quarter of 2004. However, the very deep recession of 2009 is again well captured (as is the case for the daily gap). The electricity gap seems to pick up major movements but has considerable volatility and is therefore not able to signal ordinary business cycles very well.

## Data Availability

All data are available online from the sources mentioned in the text.

## Abbreviations

COVID-19: Coronavirus disease 2019
DST: Daylight savings time
GDP: Gross domestic product
GWh: Giga-watt hour
HP: Hodrick-Prescott filter
SARS-CoV-2: Severe acute respiratory syndrome coronavirus 2
US: United States of America

## Footnotes

1 Verordnung über Massnahmen zur Bekämpfung des Coronavirus (COVID-19), https://www.admin.ch/opc/de/official-compilation/2020/573.pdf.

2 Verordnung 2 über Massnahmen zur Bekämpfung des Coronavirus (COVID-19), https://www.admin.ch/opc/de/official-compilation/2020/773.pdfand the much stronger update of March 16 here, https://www.admin.ch/opc/de/official-compilation/2020/783.pdf.

3 The statistical office of Kanton Zürich provides a number of such indicators that are freely available and potentially very useful, see https://statistik.zh.ch/internet/justiz_inneres/statistik/de/covid19.html

4 https://www.swissgrid.ch/en/home/operation/grid-data.html

5 Schweizerische Elektrizitätsbilanz, see a link on https://www.bfe.admin.ch/bfe/de/home/versorgung/statistik-und-geodaten/energiestatistiken/elektrizitaetsstatistik.html.

6 Weather data are available here, https://gate.meteoswiss.ch/idaweb/. I have experimented with average temperature of the whole day (variable tre200d0), average temperature during daytime (variable tre200j0), heating degrees (variables xhd000d0 and xno000d0), and precipitation (variable rka150d0). I use data collected from the main weather stations in Basel, Bern, Geneva, Lausanne, Lugano, Lucerne, St.Gallen, and Zürich. Weather data are updated daily.

7 Stationarity of the weather may be debatable, but not over the relatively short horizons that we analyze.

8 The HP filter minimizes a weighted sum of the deviations of the smoothed series from the original data, and the square of the curvatures of the smoothed series. The weight is controlled by one parameter, traditionally denoted with *λ*. Ravn and Uhlig (2002) have elaborated on the correct choice of *λ*. They suggest that *λ*=6.25+*p*^4^ is roughly appropriate, where *p* is a periodicity of the fluctuations that should still be contained in the smoothed series. For monthly data (*p*=12), this implies *λ*=129600; for daily data, *p*=365.25, this implies *λ*=107*e*9.

9 Number of observations: 4169, degrees of freedom: 3734, SSE: 0.7019, RMSE: 0.01371, D.W.: 2.0097.

10 Having multiple Easter-related dummies did not enhance the fit, and Easter Monday turned out to be the best choice.

11 Quarterly GDP growth data are available here, https://data.snb.ch/.
